# Steroid receptor coactivator-1: integrating steroid hormone signals to regulate brain function and disease

**DOI:** 10.3389/fnins.2026.1837573

**Published:** 2026-05-11

**Authors:** Yinghui Li, Bing Yang, Shiwei Xu, Tingting Xian, Ming Zhang

**Affiliations:** Department of Neurology, Yilong County People's Hospital, Nanchong, Sichuan, China

**Keywords:** central nervous system, learning and memory, neurodegenerative diseases, neuropsychiatric disorders, steroid receptor coactivator-1

## Abstract

Steroid receptor coactivator-1 (SRC-1), also known as nuclear receptor coactivator-1 (NCOA1), represents the first identified member of the p160 nuclear receptor coactivator family and plays a pivotal role in integrating steroid hormone signals, regulating gene transcription, and maintaining neural homeostasis in the central nervous system (CNS). SRC-1 exhibits region-specific, cell-type-specific, and sexually dimorphic expression patterns in the brain, with prominent distribution in key regions including the hippocampus, cerebral cortex, hypothalamus, and amygdala. Functional studies demonstrate that SRC-1 participates in diverse neural functions such as learning and memory, energy metabolism, emotional regulation, and reproductive behavior through modulation of synaptic plasticity-related genes, neurotrophic factors, and metabolic pathways. Aberrant SRC-1 expression is closely associated with neurodegenerative diseases, autism spectrum disorders, and glioblastoma. This review systematically summarizes the molecular structure, expression characteristics, physiological functions of SRC-1, and its roles in neurological disorders, while discussing its potential applications as a diagnostic biomarker and therapeutic target.

## 1 Introduction

Steroid hormones play crucial roles in regulating the development, functional maintenance, and plasticity of the central nervous system (CNS) (McEwen and Milner, 2017). These hormones modulate target gene transcription by binding to intracellular nuclear receptors (NRs), thereby influencing cell proliferation, differentiation, and functional status (Evans and Mangelsdorf, 2014). However, the transcriptional regulatory activity of nuclear receptors is highly dependent on the cooperative participation of coregulators, which include coactivators and corepressors. These coregulators precisely regulate the physiological effects of steroid hormones by recruiting effector molecules such as histone-modifying enzymes, chromatin remodeling complexes, and transcriptional machinery (Xu and Li, 2003).

The O’Malley research team first discovered steroid receptor coactivator-1 (SRC-1), also designated as nuclear receptor coactivator-1 (NCOA1), through yeast two-hybrid screening, representing the first cloned nuclear receptor coactivator (Oñate et al., 1995). SRC-1 belongs to the p160 nuclear receptor coactivator family, which comprises three highly homologous yet functionally distinct members: SRC-1, SRC-2, and SRC-3 (Liu et al., 2021).

In the CNS, SRC family members exhibit marked region-specific and functionally distinct expression patterns. SRC-1 is broadly expressed in the adult brain, where its region- and cell-type-specific distribution patterns underlie its diverse roles in neural function, as detailed in Section 3 of this review (Meijer et al., 2000; Nishihara et al., 2003). SRC-2 primarily regulates reproduction, lipid metabolism, and energy balance (Chopra et al., 2008), whereas SRC-3 mainly modulates hormone-dependent tumors (Varisli et al., 2023). Recent studies indicate that SRC-1 not only participates in classical steroid hormone-mediated gene transcriptional regulation but also contributes to higher neurological functions including neural development, synaptic plasticity, learning and memory, emotional regulation, and reproductive behavior through multiple signaling pathways (Nishihara et al., 2003; Bian et al., 2018; Molenda-Figueira et al., 2006). Its aberrant expression and function are closely associated with neurological disorders (Wu et al., 2020, 2021; Meng et al., 2021).

It should be noted that the preponderance of evidence reviewed herein derives from studies conducted in rodent models, principally mice and rats, and findings from these systems may not always translate directly to human biology. Nevertheless, a growing body of translational evidence is beginning to bridge this gap. Where relevant, this review highlights such translational findings alongside experimental evidence from animal models. However, the specific mechanisms of SRC-1 action in different neural circuits, functional differentiation of splice variants, synergistic/compensatory relationships with other family members, and translational value as a therapeutic target remain to be systematically elucidated.

## 2 Molecular structure and regulatory networks of SRC-1

### 2.1 Structural organization and functional domains of SRC-1

**Figure 1:**
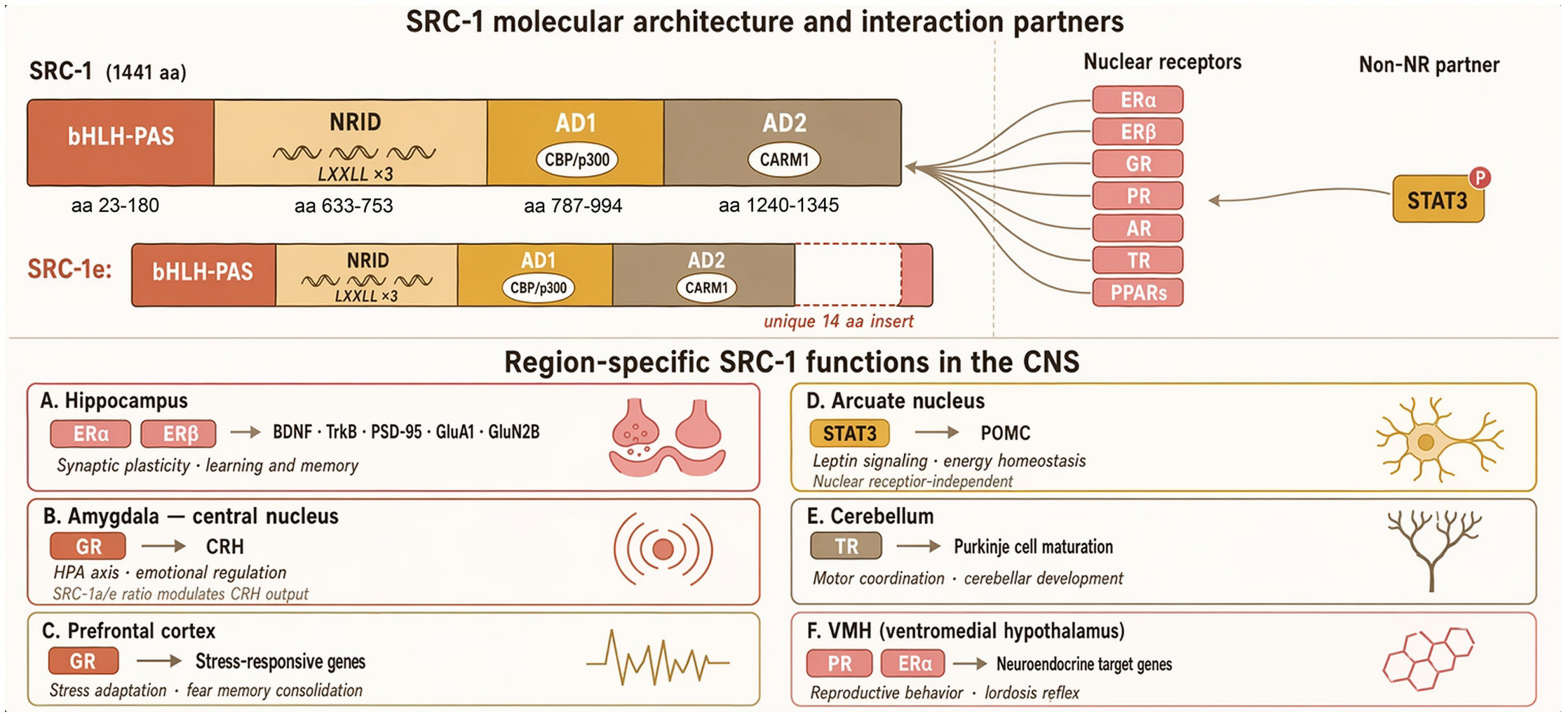
Molecular architecture, region-specific functions, and disease relevance of SRC-1 in the central nervous system. The upper panel illustrates the domain organization of SRC-1 (1,441 aa) and its isoform SRC-1e, encompassing an N-terminal bHLH-PAS domain, a central NRID harboring three LXXLL motifs, and C-terminal activation domains AD1 and AD2 that recruit CBP/p300 and CARM1, respectively. SRC-1 interacts with multiple nuclear receptors (ERα, ERβ, GR, PR, AR, TR, PPARs) and the non-NR partner phosphorylated STAT3. The lower panel depicts region-specific SRC-1 transcriptional programs across six CNS regions, including the hippocampus, amygdala, prefrontal cortex, arcuate nucleus, cerebellum, and ventromedial hypothalamus, highlighting both nuclear receptor-dependent and -independent mechanisms.

The human SRC-1 gene is located on chromosome 2p23 and encodes a protein of 1,441 amino acids with a highly conserved modular structure (Oñate et al., 1998; Chen et al., 2024). The N-terminal bHLH-PAS domain mediates interactions with transcription factors and secondary coactivators, forming multi-protein complexes (Xu et al., 2009; Daffern and Radhakrishnan, 2024). The central nuclear receptor interaction domain (NRID) contains three highly conserved LXXLL motifs, which represent the key interface for SRC-1 interaction with nuclear receptors (Xu et al., 2009). The C-terminal transcriptional activation domain comprises AD1 and AD2, which recruit CBP/p300 with histone acetyltransferase activity and histone arginine methyltransferase CARM1, respectively, promoting chromatin relaxation and transcriptional activation through histone acetylation and methylation (Spencer et al., 1997; Chen et al., 1999) (Figure 1).

In the CNS, SRC-1 engages a diverse repertoire of both nuclear receptor and non-nuclear receptor transcription factors to regulate neural gene expression programs. With respect to nuclear receptors, SRC-1 functions as a coactivator for estrogen receptors *α* and *β* (ERα, ERβ), glucocorticoid receptor, progesterone receptor, androgen receptor, thyroid hormone receptor, and peroxisome proliferator-activated receptors (PPARs), each mediating distinct aspects of brain function across specific regions and cell populations (Meijer et al., 2000; Xu et al., 2009). Beyond nuclear receptors, SRC-1 also cooperates with non-receptor transcription factors in brain-specific contexts: notably, it forms a functional complex with phosphorylated signal transducer and activator of transcription 3 (STAT3) in hypothalamic arcuate nucleus neurons to enhance proopiomelanocortin (POMC) transcription in response to leptin (Yang et al., 2019), and its bHLH-PAS domain facilitates interactions with other basic helix–loop–helix transcription factors implicated in neural differentiation and circadian rhythm regulation (Xu et al., 2009; Daffern and Radhakrishnan, 2024). At the level of secondary coactivators, the AD1 domain recruits CBP/p300, while the AD2 domain recruits CARM1, as noted above; the p/CIP coactivator has additionally been shown to cooperate with SRC-1 in regulating energy balance and brown adipose tissue development (Wang et al., 2006). Notably, however, a comprehensive and systematic characterization of the transcription factors that recruit SRC-1 to specific genomic loci in distinct neuronal subtypes, as well as the full complement of secondary coactivators mobilized by SRC-1 under different neural activity states and disease conditions, remains largely uncharacterized. Elucidating these upstream targeting determinants and downstream effector assemblies in brain-specific contexts represents a critical frontier for understanding how SRC-1 achieves its region- and cell-type-selective transcriptional outputs.

### 2.2 Splice isoforms and functional diversity

The SRC-1/NCOA1 gene generates two major functional splice isoforms, SRC-1a and SRC-1e, through alternative splicing. The latter exhibits higher expression in most cells due to deletion of 56 amino acids at the C-terminus and inclusion of a unique 14-amino acid sequence (Meijer et al., 2000, 2005; Hayashi et al., 1997). These structural differences affect transcriptional activation domain function, resulting in significant promoter-dependent and receptor-specific variations in coactivation capacity: SRC-1e demonstrates significantly stronger coactivation activity for thyroid hormone receptor, estrogen receptor, and glucocorticoid receptor on promoters containing multiple hormone response elements compared to SRC-1a (Hayashi et al., 1997; Meijer et al., 2005). In neuroendocrine regulation, alterations in SRC-1 variant ratios can selectively modulate transcriptional responses of glucocorticoid receptor target genes, influencing stress responses and fear memory consolidation (Lachize et al., 2009; Zalachoras et al., 2013, 2016), providing an important molecular mechanism for understanding differential physiological effects of steroid hormones in different brain regions (Tetel et al., 2009).

### 2.3 Post-translational modifications and activity regulation

The transcriptional coactivation function of SRC-1 is finely regulated by multiple post-translational modifications (Lonard et al., 2007; Amazit et al., 2003). Phosphorylation represents a key regulatory mechanism whereby extracellular signals (EGF, IL-6, cAMP) induce multi-site phosphorylation of SRC-1 through activation of MAPK and other kinase cascades. Specifically, ERK1/2-mediated direct phosphorylation at Thr1179 and Ser1185 enhances its interaction with nuclear receptors and ligand-dependent transcriptional activation (Rowan et al., 2000; Lopez et al., 2001). Ubiquitination modulates SRC-1 protein stability; the deubiquitinase USP4 stabilizes SRC-1 protein levels by catalyzing deubiquitination, playing a role in inflammatory regulation (Yan et al., 2025). Simultaneously, SRC-1 can inhibit the binding of E3 ubiquitin ligase SPOP to downstream target proteins, regulating their protein stability (Hong et al., 2024). Complex crosstalk exists between different modification types: histone pre-methylation can recruit p300 to enhance acetyltransferase activity, thereby promoting CARM1 methylation activity (Yi et al., 2017), enabling SRC-1 to integrate multiple signaling pathways for precise transcriptional regulation. Abnormalities in SRC-1 post-translational modifications are closely associated with tumor development and progression (Dasgupta et al., 2014) (Table 1).

**Table 1:** Post-translational modifications of SRC-1 and their functional roles.

Modification type	Major sites/enzymes	Regulatory factors/signals	Primary functions	References
Phosphorylation	Ser372, Ser395, Ser517, Ser569, Ser1033, Thr1179, Ser1185	EGF, IL-6, cAMP; ERK kinase directly phosphorylates Thr1179/Ser1185	Enhanced interaction with nuclear receptors; regulation of coactivator activity; modulation of subcellular localization.	Amazit et al. (2003) and Rowan et al. (2000)
Acetylation	Multiple sites	CBP/p300 directly acetylates SRC-1	Regulation of nuclear receptor interaction; control of coactivator complex assembly and stability.	Spencer et al. (1997)
Methylation	Arginine residues (AD2 domain)	CARM1 methyltransferase	Negative regulation of transcriptional activity; enhanced protein degradation; impaired CBP binding.	Chen et al. (1999)
Ubiquitination	Multiple sites	SPOP (E3 ubiquitin ligase); USP4 (deubiquitinase)	Control of protein stability and degradation; USP4 stabilizes SRC-1 to participate in macrophage polarization and inflammatory regulation.	Lopez et al. (2001) and Yan et al. (2025)

## 3 Expression patterns of SRC-1 in the brain

### 3.1 Regional and cell type specificity

SRC-1 exhibits highly region-specific distribution in the CNS. Immunohistochemical and *in situ* hybridization studies reveal that SRC-1 is primarily localized in neuronal nuclei, with high expression in hippocampal cornu ammonis area 1 (CA1)-CA4 pyramidal cell layers and dentate gyrus granule cell layer, cerebellar Purkinje cell layer, cerebral cortex, basolateral and central nuclei of the amygdala, paraventricular and arcuate nuclei of the hypothalamus, and basal ganglia (Meijer et al., 2000; Ogawa et al., 2001). Studies targeting splice subtypes reveal more refined distribution differences: SRC-1a exhibits relatively high expression in hypothalamic neuroendocrine nuclei, anterior pituitary, and brainstem motor nuclei, while SRC-1e is relatively enriched in the nucleus accumbens, basolateral amygdala, and certain thalamic nuclei (Meijer et al., 2000) (Table 2). This subtype-specific distribution suggests that different variants play differential regulatory roles in specific neural circuits.

**Table 2:** Expression pattern of SRC-1 in major brain regions.

Brain region	Expression level	Major splice isoforms	Primary functional associations	References
Hippocampus CA1-CA4/Dentate gyrus	+++	SRC-1a/SRC-1e	Learning and memory, synaptic plasticity	Meijer et al. (2000), Ogawa et al. (2001), and Bian et al. (2012a)
Cerebellar Purkinje cell layer	+++	SRC-1a	Motor coordination, development	Nishihara et al. (2003) and Zhang et al. (2011a)
Amygdala central nucleus/Basolateral nucleus	+++	SRC-1a (central nucleus), SRC-1e (basolateral nucleus)	Emotional regulation, stress response	Meijer et al. (2000) and Hinton et al. (2016)
Hypothalamic paraventricular nucleus/Arcuate nucleus/Ventromedial nucleus	+++	SRC-1a	Energy metabolism, HPA axis regulation, reproductive behavior	Meijer et al. (2000), Yang et al. (2019), and Picard et al. (2002)
Cerebral cortex (anterior cingulate cortex, motor cortex)	++	SRC-1a/SRC-1e	Cognitive function, motor control	Meijer et al. (2000) and Zhang et al. (2011b)
Olfactory bulb	+++	SRC-1e	Olfactory processing, neurogenesis	Meijer et al. (2000) and Misiti et al. (1999)
Nucleus accumbens	++	SRC-1e	Reward, motivation	Meijer et al. (2000)
Brainstem motor nuclei/locus coeruleus	++	SRC-1a	Motor control, arousal	Meijer et al. (2000)

+++ indicates high expression; ++ indicates moderate expression.

Regarding cell types, SRC-1 is predominantly expressed in neurons, with some expression in astrocytes, ependymal cells, and Schwann cells (Ogawa et al., 2001; Zhang et al., 2011b; Nishihara et al., 2007; Morte et al., 2018). Neural differentiation studies demonstrate that SRC-1 exhibits minimal expression in proliferating cells, significantly increases during neuronal lineage commitment, and reaches highest levels in mature neurons (Iwata and Ozawa, 2014), indicating that SRC-1 primarily participates in neuronal fate determination, differentiation, and maturation processes.

Beyond neurons, emerging evidence suggests that SRC-1 may fulfill regulatory roles in non-neuronal brain cell types, though this remains a substantially understudied area. In astrocytes, steroid hormone signaling via ERα and GR is known to modulate neuroinflammatory responses and gliotransmitter release (Meijer et al., 2000; Xu et al., 2009), and given that astrocytes express functional ERα and GR, SRC-1 may serve as a coactivator within these signaling pathways to regulate astrocyte-mediated neuroprotection and inflammatory gene expression. In oligodendrocytes, thyroid hormone receptor and PR are established regulators of myelination, both of which are known SRC-1 binding partners (Meijer et al., 2000; Xu et al., 2009), raising the possibility that SRC-1 contributes to myelin maintenance and repair through nuclear receptor coactivation in this cell type. In microglia, androgen receptor signaling influences neuroinflammatory polarization, and SRC-1’s established role as an AR coactivator (Meijer et al., 2000; Xu et al., 2009) suggests a potential modulatory function in microglial activation states. Systematic investigation of SRC-1 in these glial populations—including cell-type-specific conditional knockout models and single-cell transcriptomic profiling—is warranted to fully characterize the cellular landscape of SRC-1 function in the CNS.

### 3.2 Developmental timing and sexual dimorphism

SRC-1 expression exhibits developmental stage specificity. During mouse embryonic development, SRC-1 mRNA is widely expressed from E8.5, with highest expression in the olfactory epithelium at E14.5 and E18.5 (Misiti et al., 1999). Postnatal development shows brain region and sex dependence: female rat hippocampal SRC-1 peaks at P14, while male mice peak at P30, with expression patterns highly correlated with synaptic protein (synaptophysin, PSD-95, GluR1) developmental trajectories (Zhang et al., 2011a; Bian et al., 2012a). Cerebellar Purkinje cells peak at P7-P15, coinciding with synapse formation and functional maturation timelines (Yousefi et al., 2005). Neonatal blockade of SRC-1 reduces preoptic area volume by 46% in male rats (Auger et al., 2000), confirming its critical role in brain development and sexual differentiation. Aging significantly reduces SRC-1 expression in motor control regions, learning and memory areas, and neural stem cell-enriched regions (Zhang et al., 2011b; Matsumoto, 2002), and this age-dependent decline may participate in elderly cognitive decline and neurodegenerative diseases.

SRC-1 expression in the brain exhibits significant sexual dimorphism. In most brain regions of adult mice, male SRC-1 immunoreactivity is significantly higher than in females, particularly in learning and memory, motor control, and reproduction-related nuclei, with SRC-1’s regional and sex-specific distribution patterns consistent with certain steroid receptors (Bian et al., 2011). In the male hippocampus, orchidectomy leads to decreased SRC-1 expression, which can be dose-dependently restored by testosterone supplementation (Qiu et al., 2016). Mechanistic studies reveal that hippocampal neurons convert testosterone to estradiol (E2) via aromatase, with locally synthesized E2 exerting strong stimulatory effects on SRC-1. After aromatase inhibitor letrozole blocks E2 synthesis, hippocampal SRC-1 expression decreases, accompanied by abnormal actin polymerization, synapse loss, and spatial memory impairment (Zhao et al., 2017, 2018). Letrozole treatment causes more severe cognitive deficits than orchidectomy, and the effects of testosterone and aromatase overexpression can be blocked by SRC-1 inhibition (Zhao et al., 2018), demonstrating that SRC-1 plays a pivotal role in mediating local E2 regulation of hippocampal function. In contrast, ovariectomy effects on female hippocampal SRC-1 appear only transiently at 2 weeks post-surgery (Bian et al., 2012b), suggesting female hippocampal SRC-1 regulation depends more on local neurosteroid synthesis than circulating hormones.

### 3.3 Functional complementarity between SRC-1 and family members

Partial functional complementarity exists among SRC family members, though compensation degree is highly dependent on expression levels of each member in specific brain regions. In SRC-1^−/−^ mouse cerebellum, mild SRC-2 upregulation correlates with recovery timeline from delayed Purkinje cell development (Nishihara et al., 2003). However, in the olfactory bulb, SRC-1 loss does not induce compensatory upregulation of other members. In testicular tissue, despite high SRC-2 and low SRC-1 expression, only SRC-2^−/−^ mice exhibit spermatogenesis defects (Gehin et al., 2002), suggesting low-expressing members cannot fully compensate. More severely, most SRC-1/SRC-2 double knockout mice die at birth (Mark et al., 2004), with surviving double heterozygotes showing significantly enhanced thyroid hormone resistance (Weiss et al., 2002), indicating certain physiological functions require threshold SRC family member levels rather than single member dependence.

Differences between acute and chronic deletion experiments further reveal compensation mechanism complexity. Acute antisense oligonucleotide interference with SRC-1 inhibits hypothalamic estrogen-induced progesterone receptor synthesis and sexual behavior in wild-type mice but not in SRC-1^−/−^ mice, while SRC-2 antisense oligonucleotides are effective in both genotypes (Apostolakis et al., 2002), suggesting genetic SRC-1 deletion permits developmental adaptive SRC-2 upregulation. These findings collectively demonstrate that the compensatory capacity of SRC family members is highly context-dependent, varying with tissue type, developmental stage, and the nature of genetic manipulation.

## 4 Functions of SRC-1 in the central nervous system

### 4.1 Learning, memory, and synaptic plasticity

Hippocampus-specific SRC-1 knockdown reveals its critical role in learning and memory. Morris water maze testing shows that knockdown mice exhibit prolonged escape latency, reduced time spent in target quadrant, and decreased platform crossings (Bian et al., 2018). In object recognition tasks, long-delay (24-h) memory is impaired while short-term memory (1 h) remains relatively normal (Bian et al., 2018). Contextual fear conditioning studies demonstrate that hippocampal SRC-1 expression is time-dependently upregulated after training; SRC-1 knockdown significantly impairs contextual fear memory consolidation and reconsolidation but has minimal effect on cued fear memory (Chen X. et al., 2020), reflecting its brain region-specific action.

SRC-1 regulation of hippocampal synaptic plasticity represents the core mechanism underlying its influence on learning and memory. Knockdown leads to reduced long-term potentiation (LTP) amplitude and shortened maintenance in the CA1 region, accompanied by decreased expression of synaptic proteins including synaptophysin, PSD-95, GluA1, and GluN2B (Bian et al., 2018, 2012a). Electron microscopy analysis reveals approximately 30% reduction in synapse number and thinning of postsynaptic density (Bian et al., 2018). At the molecular level, CREB phosphorylation decreases after LTP induction (Bian et al., 2018), and reduced BDNF levels attenuate TrkB downstream signaling pathways (Bian et al., 2014). SRC-1 primarily mediates estrogen’s transcriptional activation of synaptic plasticity genes through ERα, with its functional loss weakening estrogen’s regulation of hippocampal cognitive function (Tetel, 2009), providing a molecular basis for understanding postmenopausal cognitive decline in women. Specifically, at the molecular level, estradiol acting through ERα and ERβ recruits SRC-1 to the promoters of synaptic plasticity genes in hippocampal neurons, including those encoding BDNF and its receptor TrkB, thereby enhancing transcriptional output in an AF-2 domain-dependent manner (Hong et al., 2024; Bian et al., 2014; Tetel, 2009). This ERα/ERβ–SRC-1 transcriptional complex represents the canonical nuclear receptor–coactivator mechanism through which gonadal hormones modulate hippocampal synaptic strength and memory consolidation.

Moreover, recent studies reveal that SRC-1 regulates actin dynamics through the PI3K/Rictor/mTORC2 pathway, affecting Cofilin and Profilin-1 expression and thereby modulating dendritic spine formation and maintenance (Zhao et al., 2017, 2018). These studies demonstrate that SRC-1 integrates sex hormone signals and neuronal activity-dependent transcriptional programs, playing critical roles at multiple levels of synaptic structure, function, and plasticity, though precise spatiotemporal regulatory mechanisms in different learning stages require further investigation.

### 4.2 Energy metabolism regulation

SRC-1 plays a central role in hypothalamic energy homeostasis regulation. SRC-1 knockout mice exhibit progressive obesity with 15–25% increased adult body weight, primarily due to reduced energy expenditure (Picard et al., 2002). Metabolic monitoring reveals decreased oxygen consumption rate and carbon dioxide production rate, disrupted circadian rhythm of respiratory exchange ratio, and weakened body temperature maintenance capacity during cold exposure (Picard et al., 2002). Mechanistically, SRC-1 enhances POMC transcription in arcuate nucleus POMC neurons through interaction with phosphorylated STAT3. POMC neuron-specific SRC-1 deletion leads to attenuated leptin-induced depolarization, downregulated POMC expression, and high-fat diet-induced obesity (Yang et al., 2019). Furthermore, the developmental arrest of brown adipose tissue and the impairment of adaptive thermogenesis observed in p/CIP and SRC-1 double knockout mice provide direct functional evidence supporting SRC-1’s essential role in adipose tissue energy metabolism. It is noteworthy that, in contrast to the canonical nuclear receptor coactivation model, the SRC-1–STAT3 interaction in arcuate nucleus POMC neurons represents a non-nuclear-receptor mechanism of SRC-1 action, wherein SRC-1 is recruited not by a steroid hormone–bound nuclear receptor but by cytokine-activated STAT3 (Yang et al., 2019). This dual capacity of SRC-1 to serve both nuclear receptor-dependent and nuclear receptor-independent transcriptional roles within the CNS underscores the functional versatility of this coactivator beyond its classical designation.

As a coactivator for multiple nuclear receptors (PPARs, TR, ER, ERR), SRC-1 occupies a central position in metabolic networks (Dasgupta et al., 2014). Neonatal studies demonstrate that thyroid hormone can regulate SRC-1 expression in a region-specific manner (Iannacone et al., 2002), suggesting its participation in neuroendocrine developmental regulation. In summary, SRC-1 coordinates energy balance by integrating central leptin signaling and peripheral tissue metabolism; its dysfunction leads to metabolic syndrome, though specific mechanisms in different neuronal populations and metabolic tissues require elucidation.

### 4.3 Emotional regulation and stress response

SRC-1 is highly expressed in the central and medial nuclei of the amygdala (Bian et al., 2011; Hinton et al., 2016) and participates in HPA axis regulation as a glucocorticoid receptor coactivator (Lachize et al., 2009). Knockout mice show reduced basal corticotropin-releasing hormone (CRH) mRNA in the central amygdala and attenuated hypothalamic CRH upregulation after chronic stress (Lachize et al., 2009). By modulating SRC-1 splice variant ratios, glucocorticoid-induced CRH expression can be significantly altered, affecting contextual fear memory consolidation with minimal impact on cued fear, demonstrating brain region specificity (Zalachoras et al., 2016).

Mechanistically, the glucocorticoid receptor serves as the principal nuclear receptor mediating SRC-1 recruitment within the HPA axis. Upon cortisol or corticosterone binding, the ligand-activated GR–SRC-1 complex binds to glucocorticoid response elements in the promoters of CRH and other stress-responsive genes, modulating their transcriptional activity in a region-specific manner in the hypothalamus and prefrontal cortex (Xu et al., 2009; Lachize et al., 2009; Zalachoras et al., 2016). Disruption of this GR–SRC-1 interaction may therefore represent a key mechanistic node linking impaired stress hormone signaling to the heightened emotional vulnerability observed in SRC-1-deficient animals.

Stress-induced impairment of neuroplasticity represents a core pathological mechanism of depression. After chronic unpredictable mild stress (CUMS) exposure, hippocampal and prefrontal cortex SRC-1 expression is downregulated, and SRC-1 gene knockout mice exhibit higher susceptibility to CUMS-induced depressive-like behaviors, suggesting SRC-1’s protective role in stress adaptation (Wu et al., 2021). These findings indicate that SRC-1 achieves fine regulation of glucocorticoid signaling through splice variant switching, providing intervention targets for stress-related disorders, though dynamic mechanisms of splicing transitions under stress require further exploration.

### 4.4 Reproductive behavior

SRC-1 plays a critical role in sex hormone-dependent reproductive behavior. Neonatal hypothalamic injection of SRC-1 antisense oligonucleotides blocks estrogen-induced brain defeminization, causing genetic males to exhibit feminized characteristics such as reduced sexually dimorphic nucleus of the preoptic area (SDN-POA) volume and impaired adult male reproductive behavior (Auger et al., 2000), demonstrating that SRC-1 is a necessary molecule mediating sex hormone organizational effects.

In adult females, reducing SRC-1 and CBP levels in the ventromedial nucleus of the hypothalamus disrupts ER-mediated PR expression, impairing lordosis reflex and proactive courtship behaviors (hopping, darting, ear wiggling) (Molenda-Figueira et al., 2006; Molenda et al., 2002), indicating that coactivators synergistically integrate complex reproductive behavioral patterns across different neuroendocrine pathways. However, although SRC-1 global knockout mice exhibit partial hormone resistance, both males and females maintain fertility (Xu et al., 1998), likely attributable to compensatory upregulation of family members such as TIF2 (transcriptional intermediary factor 2, also known as SRC-2/NCOA2) (Xu et al., 1998). The nuclear receptor basis of this regulation involves progesterone receptor and ERα acting in concert: ligand-bound PR and ERα recruit SRC-1 to the promoters of neuroendocrine target genes within the ventromedial nucleus of the hypothalamus and the SDN-POA, directly linking circulating gonadal hormone levels to the transcriptional programs governing reproductive behavior (Dasgupta et al., 2014; Auger et al., 2000; Xu et al., 1998). The dependence of lordosis behavior on SRC-1 thus exemplifies the classical nuclear receptor coactivation model operating within a discrete neural circuit. These studies demonstrate that SRC-1 is a key mediator of sex hormone organizational and activational effects, though molecular bases of compensatory mechanisms and time-specific functions require deeper investigation.

### 4.5 Other brain functions

Beyond roles in emotional and reproductive regulation, SRC-1 exhibits expression and potential functions in multiple brain functional systems. Regarding motor function, SRC-1’s role in cerebellar development has been well established (Nishihara et al., 2003). Immunohistochemical studies show that SRC-1 expression in cerebellar Purkinje cells exceeds other SRC family members. Adult SRC-1 knockout mice demonstrate moderate motor dysfunction in standard motor coordination tests including rotarod and balance beam (Nishihara et al., 2003). Regarding olfactory system development, SRC-1 transcript expression is highest in the olfactory epithelium at embryonic stages E14.5 and E18.5 (Misiti et al., 1999), suggesting possible participation in olfactory system formation and development.

## 5 SRC-1 and neurological disorders

### 5.1 Cognitive impairment and neurodegenerative diseases

SRC-1 plays a critical role in hippocampus-dependent learning, memory, and synaptic plasticity. As described above (Section 4.1), SRC-1 regulates synaptic plasticity and BDNF–TrkB signaling in the hippocampus (Bian et al., 2018); this function is of particular relevance to AD pathophysiology, given the well-established synaptic deficits that characterize early-stage disease (Tetel, 2009; Pike et al., 2009).

Mechanistic studies reveal that SRC-1 regulates actin cytoskeleton dynamics and dendritic spine morphology through the estrogen receptor-mTORC2 pathway (Zhao et al., 2018). The aromatase inhibitor letrozole reduces brain estrogen synthesis, leading to decreased SRC-1 expression and subsequent hippocampal synaptic protein reduction and cognitive impairment (Liu et al., 2015). Notably, Wu et al. (2020) found in APP/PS1 mouse models that SRC-1 gene knockout had no significant effects on *β*-amyloid (Aβ) deposition, glial activation, or synaptic protein expression, suggesting SRC-1’s cognitive protective effects may be independent of classical Aβ pathology and more related to synaptic function maintenance and neuroplasticity regulation.

Aging-associated cognitive decline is closely related to SRC-1 expression downregulation. Liu et al.’s (2025) recent study analyzed GTEx database data, finding significantly lower SRC-1/NCOA1 expression in hippocampus and hypothalamus of elderly compared to young populations. Animal experiments confirm that SRC-1 knockout mice and mice carrying humanized mutations (L1376P) exhibit contextual memory deficits and impaired hippocampal CA1 neuronal plasticity at 6 months of age. Mechanistic studies reveal that SRC-1 significantly upregulates neuroprotective protein S100A6 expression through transcriptional regulation, suggesting it may delay cognitive decline through integrated sex hormone signaling and neuroprotective mechanisms. These findings indicate that SRC-1 may play a core role in maintaining cognitive function and delaying aging-related cognitive decline through integrated sex hormone signaling and neuroprotective mechanisms.

In Parkinson’s disease (PD), sex steroid hormones’ protective effects on dopaminergic neurons have been widely reported, potentially related to sex-differential PD incidence rates (Bourque et al., 2009). Estrogen promotes dopaminergic neuron survival by activating PI3K/Akt and MAPK signaling pathways. Considering SRC-1’s critical position in mediating sex hormone actions, it is predicted to participate in sex hormone-mediated protective effects on dopaminergic neurons, though direct experimental evidence is currently lacking.

### 5.2 Mood and psychiatric disorders

Stress-induced neuroplasticity impairment and hypothalamic–pituitary–adrenal (HPA) axis dysfunction represent core pathological mechanisms of depression. The observation that SRC-1-deficient mice exhibit heightened susceptibility to chronic unpredictable mild stress (CUMS)-induced depressive-like behavior, as discussed in Section 4.3 (Wu et al., 2021), implicates impaired SRC-1-mediated glucocorticoid and estrogen signaling as a potential mechanistic contributor to depression vulnerability (Wu et al., 2021). Moreover, depression exhibits significant sex differences, with female prevalence approximately double that of males (Otte et al., 2016), indicating sex hormone roles in depression pathogenesis. Estrogen exerts antidepressant effects by regulating synaptic plasticity, neurotrophic factor expression, and neuroprotection (Gillies and McArthur, 2010). As a key coactivator of estrogen receptors (Bian et al., 2014), SRC-1 represents an important molecular mediator linking sex hormone signaling and emotional regulation.

Mechanistic studies indicate that hippocampal neuroplasticity impairment is an important pathological feature of depression (Serafini et al., 2014). Hippocampally synthesized estradiol regulates brain-derived neurotrophic factor (BDNF) and synapse-related gene expression, while SRC-1 is highly expressed in the hippocampus and developmentally correlated with key synaptic proteins (Bian et al., 2014), suggesting SRC-1 participates in estrogen-mediated hippocampal plasticity regulation, though specific mechanisms require further investigation. Additionally, CRH plays important roles in depression and anxiety regulation. SRC-1 is expressed in the hypothalamic paraventricular nucleus and amygdala, participating in estrogen-induced CRH expression regulation (Lalmansingh and Uht, 2008). Animal studies show that chronic stress-induced CRH expression upregulation is significantly attenuated in SRC-1 knockout mice (Lachize et al., 2009), indicating SRC-1’s important role in HPA axis regulation in stress-related psychiatric disorders.

Autism spectrum disorder (ASD) exhibits significant sex differences (male prevalence approximately 4-fold higher than females), prompting researchers to focus on sex hormone signaling pathway roles. Crider et al.’s study of middle frontal gyrus tissue from 13 ASD patients and 13 controls revealed systematic abnormalities in estrogen signaling pathways in ASD patient brain tissue: SRC-1 mRNA expression decreased 34%, aromatase (CYP19A1) decreased 38%, ERβ decreased 35%, while ERα showed no significant changes (Crider et al., 2014). Other estrogen receptor coactivators such as CBP (decreased 77%) and P/CAF (decreased 52%) also exhibited significant downregulation, while corepressors and other p160 family members (TIF-2, AIB-1) remained unchanged, displaying a specific alteration pattern. These molecular changes showed significant positive correlations, supporting the hypothesis of synergistic dysfunction of the estradiol/ERβ/SRC-1 pathway in ASD brains (Crider et al., 2014).

### 5.3 SRC-1 and brain tumors

SRC-1 plays important tumor-promoting roles in glioblastoma (GB) development and progression. Gong et al. (2021) found that SRC-1 expression levels positively correlate with pathological grade and negatively correlate with patient prognosis. Functional studies reveal that SRC-1 not only promotes GB cell proliferation, migration, and tumor growth but also enhances GB stem cell-like characteristics through post-transcriptional regulation of long non-coding RNA XIST via the XIST/miR-152/KLF4 signaling axis (Gong et al., 2021). Tumor angiogenesis is a key process in GB malignant progression. Zhang and Shi (2019) discovered that SRC-1 is upregulated in glioma vasculature and significantly enhances basic fibroblast growth factor (bFGF)-mediated angiogenesis. These studies demonstrate that SRC-1 promotes glioma malignant progression by regulating GB stem cell characteristics and tumor angiogenesis, providing potential molecular targets for GB therapy.

### 5.4 SRC-1 and brain trauma

SRC-1 expression changes in stress-related brain regions are closely associated with post-traumatic stress disorder (PTSD)-like behaviors. Whitaker et al. (2016) established a rat PTSD model using predator odor-paired context, finding that rats exhibiting avoidance behavior showed decreased SRC-1 expression in the hypothalamic paraventricular nucleus and central amygdala but increased expression in the ventral hippocampus, with SRC-1 expression levels significantly correlating with avoidance behavior. Pre-stress corticosterone pretreatment reduced stress-induced avoidance behavior incidence and severity, suggesting HPA axis functional status may participate in stress-related neural adaptation through altered SRC-1 expression.

**Figure 2:**
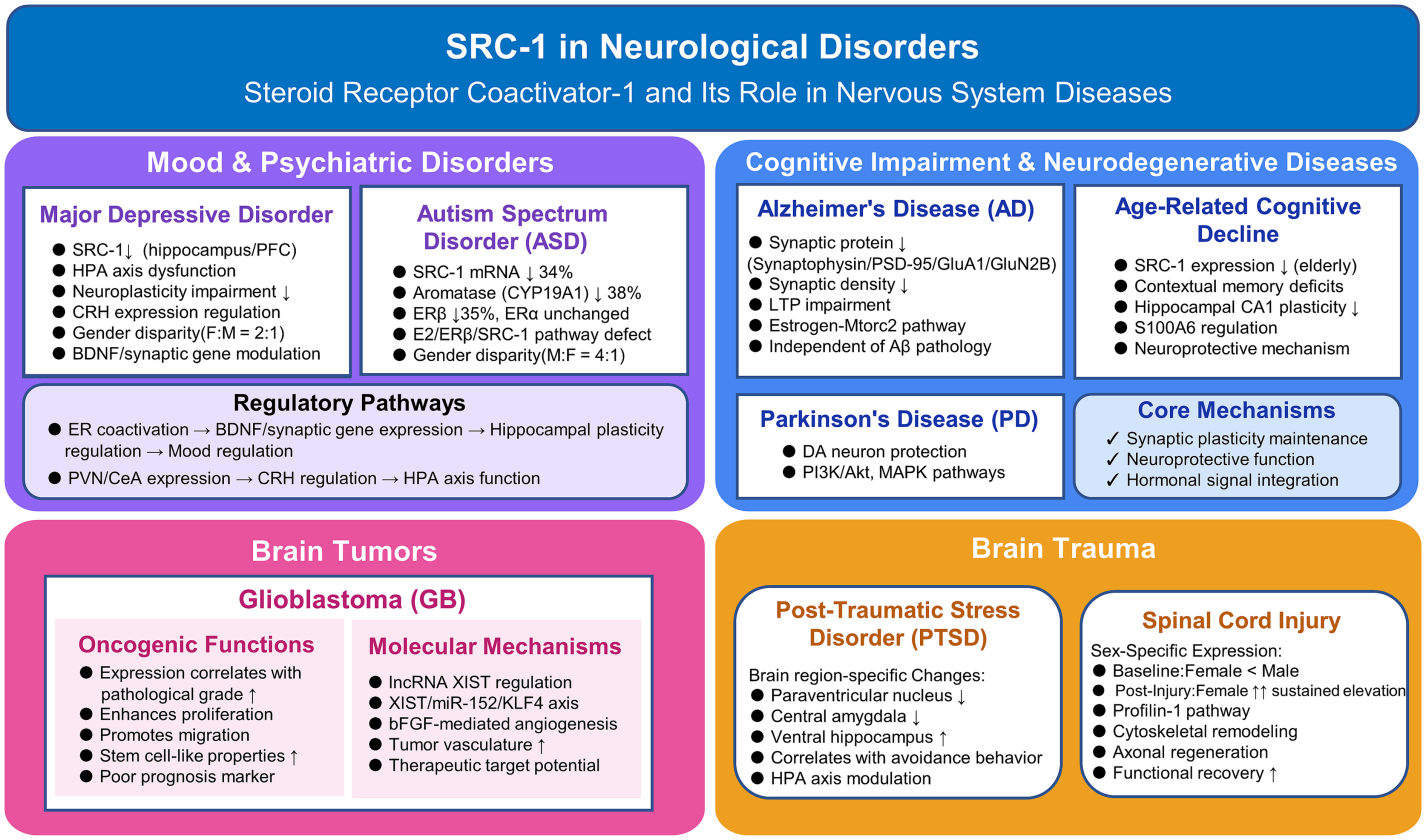
Roles of SRC-1 in neurological disorders. SRC-1 regulates multiple neurological conditions including mood and psychiatric disorders (major depressive disorder, autism spectrum disorder), cognitive impairment and neurodegenerative diseases (Alzheimer’s disease, Parkinson’s disease, age-related cognitive decline), brain tumors (glioblastoma), and brain trauma (post-traumatic stress disorder, spinal cord injury) through diverse molecular mechanisms involving synaptic plasticity, neuroprotection, and hormonal signaling pathways.

Clinical studies reveal significant sex differences following central nervous system injuries, with females often demonstrating superior neurological recovery compared to males (Zhang et al., 2010; Herson et al., 2009). Xiao et al. discovered sex-specific SRC-1 expression patterns in spinal cord injury mouse models (Xiao et al., 2017). Under normal conditions, female mice exhibit lower SRC-1 expression than males. However, after spinal cord injury, female SRC-1 expression significantly increases and persists longer, remaining higher than males at 1 and 3 days post-injury (Xiao et al., 2017). This finding suggests that increased SRC-1 expression after female spinal cord injury may promote neurological recovery by enhancing estrogen’s regulation of Profilin-1-mediated cytoskeletal remodeling and axonal regeneration (Figure 2).

## 6 Perspectives and conclusions

SRC-1, as the first member of the p160 nuclear receptor coactivator family (Xu and Li, 2003), possesses modular functional domain structure and multiple splice isoforms that regulate steroid hormone-dependent gene transcription. Its post-translational modification network further enhances functional diversity (Lonard et al., 2007; Amazit et al., 2003; Rowan et al., 2000; Lopez et al., 2001; Yi et al., 2017; Dasgupta et al., 2014). SRC-1 exhibits significant region-specific, cell type-specific, and sexually dimorphic expression in the brain (Meijer et al., 2000; Zhang et al., 2011a,b; Misiti et al., 1999; Bian et al., 2011, 2012a; Yousefi et al., 2005), participating in neurological functions including learning and memory, energy metabolism, emotional regulation, and reproductive behavior (Bian et al., 2014, 2018; Molenda-Figueira et al., 2006; Lachize et al., 2009; Zalachoras et al., 2016; Chen X. et al., 2020; Tetel, 2009; Picard et al., 2002; Iannacone et al., 2002; Hinton et al., 2016; Molenda et al., 2002; Xu et al., 1998). Its aberrant expression is associated with neuropathological diseases including Alzheimer’s disease, depression, autism spectrum disorders, and brain tumors (Wu et al., 2020, 2021; Liu et al., 2025; Crider et al., 2014; Gong et al., 2021; Zhang and Shi, 2019).

Despite significant research progress, key scientific questions remain for deeper investigation. First, functional specificity of SRC-1 splice variants and their regulatory mechanisms in different neural circuits require systematic elucidation. Zalachoras et al.’s (2016) study demonstrated that altering the SRC-1a to SRC-1e ratio in the central amygdala through antisense oligonucleotide technology significantly affects glucocorticoid-induced CRH expression and anxiety-like behaviors, suggesting selective variant regulation may become a potential strategy for stress-related disorder intervention. Second, SRC-1 expression in neuronal cytoplasm and nerve fibers (Zhang et al., 2011b; Bian et al., 2011) suggests possible participation in rapid non-genomic signal transduction, though specific molecular mechanisms remain unclear. Third, SRC-1’s roles in neurodegenerative diseases exhibit complexity and stage dependence. Liu et al.’s (2025) research reveals that SRC-1 delays aging-related cognitive decline through transcriptional regulation of neuroprotective protein S100A6, while Wu et al. (2020) found embryonic global SRC-1 knockout had no significant effect on *β*-amyloid (Aβ) deposition in APP/PS1 mice. This difference reflects that SRC-1’s cognitive protective effects depend more on synaptic function maintenance and neuroplasticity regulation rather than direct intervention in amyloid pathology; however, embryonic global gene knockout may trigger developmental compensatory mechanisms, limiting accurate assessment of SRC-1’s roles in disease progression. These findings provide new perspectives for developing intervention strategies targeting early synaptic dysfunction-related cognitive decline.

Before SRC-1 can be realistically advanced as a viable therapeutic target in neurological disease, several fundamental mechanistic questions must be resolved. First, what is the full identity of the transcription factors that direct SRC-1 recruitment to specific genomic loci in different neuronal populations, and how do pathological states such as neurodegeneration or chronic stress alter this targeting specificity? Second, how do the secondary coactivators mobilized by SRC-1’s AD1 and AD2 domains—particularly CBP/p300 and CARM1—integrate with upstream signaling cascades in neural circuits, and which of these protein–protein interactions are sufficiently selective to permit pharmacological intervention without disrupting broadly essential transcriptional processes? Third, given evidence that SRC-1 is present in neuronal cytoplasm and axonal processes (Zhang et al., 2011b; Bian et al., 2011), to what extent do transcription-independent, non-genomic functions of SRC-1 contribute to its neural effects, and how do genomic and non-genomic modes of action interact to shape synaptic and behavioral outcomes? Addressing these mechanistic gaps is a prerequisite for moving from correlative expression studies to rational, mechanism-based therapeutic design targeting the SRC-1 axis.

SRC-1 as a potential therapeutic target faces numerous challenges and opportunities. Given functional redundancy and compensatory mechanisms among SRC family members (Gehin et al., 2002; Mark et al., 2004; Apostolakis et al., 2002), future therapeutic strategies should emphasize functionally selective regulation or targeted intervention of specific splice variants rather than complete inhibition of single members. Considering SRC-1 expression’s significant sex differences and hormone dependence (Bian et al., 2011, 2012b; Qiu et al., 2016; Zhao et al., 2017, 2018), combined strategies of hormone replacement therapy and SRC-1 functional regulation may have synergistic effects in preventing postmenopausal cognitive impairment in women, though effectiveness and safety require rigorous clinical trial validation (Morrison et al., 2006; Henderson, 2014). The mechanism by which selective estrogen receptor modulators (SERMs) exert neuroprotective effects through influencing SRC-1 recruitment deserves in-depth exploration (Zhao et al., 2015). Additionally, new technology applications will open new avenues for SRC-1 research: single-cell sequencing and spatial transcriptomics can resolve SRC-1 expression patterns and functional heterogeneity in different neuronal subtypes; genome-wide association studies (GWAS) can systematically identify genetic associations between NCOA1 gene variants and neuropsychiatric diseases (Jansen et al., 2019), helping identify high-risk populations and explore early prevention strategies (Morrison et al., 2006).

From a translational research perspective, advancing SRC-1 research requires addressing the following key questions. First, more specific and selective SRC-1 functional regulatory tools need development, including small molecules or biological macromolecules targeting specific splice variants and functional domains. Second, the clinical value of SRC-1 expression levels, splicing patterns, and post-translational modification states as biomarkers for neurodegenerative diseases requires systematic evaluation through large-scale prospective cohort studies. Third, personalized treatment strategies based on NCOA1 genotypes, particularly precise applications in hormone replacement therapy, need more evidence-based medicine support (Morrison et al., 2006; Henderson, 2014). Fourth, in neurological tumor treatment, elucidating molecular mechanisms by which SRC-1 regulates tumor stemness and angiogenesis (Gong et al., 2021; Zhang and Shi, 2019) may provide new theoretical foundations for optimizing immunotherapy.

In summary, SRC-1 plays a central role in the central nervous system by integrating multiple signaling pathways and maintaining neurological homeostasis. In-depth understanding of SRC-1’s action mechanisms and regulatory networks will provide important theoretical foundations for prevention and treatment of neurodegenerative diseases, psychiatric disorders, and glioblastoma. Future research should prioritize conditional gene manipulation technologies to resolve SRC-1’s spatiotemporal-specific and cell type-specific functions (Saunders et al., 2018), utilize high-throughput omics technologies to systematically map its transcriptional regulatory networks and interacting proteomes (Armand et al., 2021; Chen W. T. et al., 2020), and conduct large-scale human genetic and epigenetic studies to establish genotype–phenotype-environment interaction associations (Yang et al., 2019; Crider et al., 2014; Jansen et al., 2019). With the development of precision medicine and systems biology, multilevel integrative analysis based on SRC-1 functional status, genetic variants, and epigenetic modifications is expected to provide scientific evidence for formulating individualized diagnostic and therapeutic strategies (Livingston et al., 2020; Barabási et al., 2011).
